# Elastic scaffolds reinforced stem cell-laden collagen-derived hybrid hydrogels to engineer 3D anisotropic cellular microenvironment

**DOI:** 10.1016/j.mtbio.2026.103385

**Published:** 2026-06-22

**Authors:** Hongjuan Weng, Lei He, Wen Chen, Vahid Ansari, Sabine van Rijt, Monize C. Decarli, Katrien V. Bernaerts, Lorenzo Moroni

**Affiliations:** a Complex Tissue Regeneration Department, MERLN Institute for Technology-Inspired Regenerative Medicine, Maastricht University, the Netherlands; b Sustainable Polymer Synthesis Group, Aachen-Maastricht Institute for Biobased Materials, Maastricht University, the Netherlands; c Instructive Biomaterials Engineering Department, MERLN Institute for Technology-Inspired Regenerative Medicine, Maastricht University, the Netherlands; d Department of Biomaterials and Biomedical Technology, University Medical Center Groningen, University of Groningen, the Netherlands

**Keywords:** Soft-hard hybrid scaffold, Nanocomposite collagen-derived hydrogels, poly(ester amide) scaffolds, Cell alignment, Biochemical cues, Biophysical anisotropic cue

## Abstract

Replicating complex, robust and organized three-dimensional (3D) cellular microenvironments that exist in many human tissues is essential for understanding the cell-matrix interactions and paving the foundation for tissue engineering. However, biofabrication strategies for *in vitro* modeling such complex, mechanically robust, and 3D anisotropic cell networks by cell-laden hydrogel-scaffolds are far less developed. This work describes 3D anisotropic soft-hard hybrid scaffolds with either single macroscale anisotropy or dual macro-micro-scale anisotropy by incorporating collagen-derived hydrogels with human mesenchymal stem cells (hMSCs) and 3D printed poly(ester amide) (PEA) scaffolds with uniaxial architecture. As a hard scaffold, the uniaxial PEA scaffold endows hybrid constructs with macroscale anisotropy, good elasticity, and mechanical properties. As a soft matrix, methacrylated collagen peptide (COPMA) hydrogel endows hybrid constructs with rapid light response, leading to *in situ* formation of 3D cellular networks. Magnetic nanoparticles (MNPs) laden COPMA hydrogel endows hybrid constructs with rapid dual light-magnetic response and magnetic-driven dual macro-micro-scale anisotropy. PEA-COPMA constructs act as biocompatible biochemical cues to induce the encapsulated hMSCs to attach and spread between the scaffold and the hydrogel. These encapsulated hMSCs showed improved cell spreading, alignment and differentiation in the dual macro-micro-scaled aligned PEA-COPMA-MNP constructs due to the combination of biochemical and biophysical anisotropic cues. This hybrid manufacturing strategy, which directly incorporates light-magnetic-responsive cell-laden nanocomposite hydrogels with elastic organized scaffolds, shows great potential in developing advanced mechanically reinforced 3D organized *in vitro* cellular microenvironments.

## Graphical abstract

**Figure:**
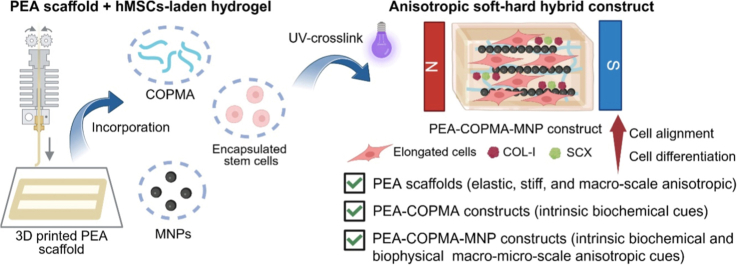

## 1 Introduction

Many human tissues, such as ligaments, tendons, and muscles, are composed of highly organized cells embedded within a mechanically reinforced, anisotropic extracellular matrix (ECM) [1,2]. Importantly, these native musculoskeletal tissues exhibit hierarchical anisotropic organization across multiple length scales, ranging from aligned collagen fibrils and cellular orientation at the microscale (∼several micrometers to tens of micrometers) to aligned fascicles and tissue architectures at the macroscale (∼hundreds of micrometers to millimeters) [3,4]. These hierarchically anisotropic, mechanically robust constructs are critical for their functions, including directional signal conduction, cell spreading, and load bearing [5,6]. Engineering biomimetic constructs as *in vitro* models to reproduce such complex cell networks has emerged as a critical focus in tissue engineering [[7], [8], [9]]. However, replicating these features remains challenging, especially systematically achieving a mechanically robust, hierarchically anisotropic 3D structure with biocompatibility in one construct.

3D printing techniques can endow thermoplastic polymers with robust mechanical property and macroscale uniaxial morphology, but struggle to induce *in situ* 3D cell alignment in microscale [10,11]. Common 3D printing techniques include photopolymerization- and extrusion-based 3D printing. Photopolymerization-based 3D printing techniques, including stereolithography (SLA), digital light processing (DLP), liquid crystal display (LCD), and volumetric printing, enable high-resolution 3D printing of scaffolds through spatially controlled photopolymerization [[12], [13], [14], [15]]. However, these technologies are typically limited by material selection (low-viscosity photopolymerizable inks), the potential cytotoxicity of photoinitiator or solvents, and relatively brittle mechanical properties [16]. In contrast, extrusion-based printing techniques show broader material compatibility. Extrusion-based printing, including fused deposition modeling (FDM), melt electrowriting (MEW) and direct ink writing (DIW), can directly process high-viscosity polymers and thermoplastics in molten or solution states into 3D-printed scaffolds [17]. Among them, FDM is particularly suitable for direct printing molten thermoplastics into mechanically robust and elastic scaffolds without the need for dissolution in potentially cytotoxic solvents [18]. For example, Aboal-Castro et al. 3D printed poly(*ϵ*-caprolactone) (PCL) scaffolds by FDM with an ultimate tensile stress of more than 10 MPa [19]. They observed enhanced cell alignment along aligned microgrooves on the surface of PCL scaffolds via the laser-engraved technique [19]. Although 3D printed scaffolds could induce cell orientation along the surface of organized filaments, they typically require post-cell seeding, and the cell spreading could be disordered between adjacent filaments in long-term cultures. Thus, there is a need to develop *in situ* 3D anisotropic microenvironments that eliminate the need for post-processing while possessing long-term stable aligned cell networks.

Cell-laden hydrogels have been widely investigated for *in situ* 3D cell encapsulation and proliferation, allowing homogenous cell distribution and inducing cell-mediated ECM remodeling *in vitro* [20]. Furthermore, anisotropic cell-laden hydrogels can generate *in situ* microscale aligned cellular networks through external physical stimulation, including mechanosensing [21], light [22], sound [23], and magnetism [24]. Among these strategies, magnetic-responsive hydrogels incorporating iron oxide magnetic nanoparticles (MNPs) have attracted considerable attention due to their ability to rapidly generate anisotropic architectures under external magnetic fields [[25], [26], [27]]. The incorporation of aligned nanoparticles can induce microscale matrix anisotropy and direct cellular orientation within the hydrogel network [28]. Although anisotropic cell-laden hydrogels can mimic microscale anisotropic ECM environments, most of them lack sufficient mechanical reinforcement for load-bearing tissue engineering applications.

To overcome these limitations, hybrid systems combining uniaxial hard scaffolds and anisotropic cell-laden soft hydrogels could be a promising strategy for fabricating mechanically reinforced anisotropic hybrid constructs. However, the existing hybrid scaffolds face challenges to achieve 3D anisotropic cellular networks throughout both the scaffold and hydrogel regions [29]. For example, Spauwen et al. combined the anisotropic melt electrowritten PCL scaffolds with methacrylated gelatin (GelMA) hydrogels, but cell networks only aligned along the filaments of the scaffold, not in the GelMA hydrogel region [30].

Among the reported soft-hard hybrid scaffolds for tissue engineering, many studies choose biodegradable elastic polymers (e.g., PCL and polylactide) as scaffold sources and incorporate them with natural hydrogels (e.g., GelMA and methacrylated hyaluronic acid) [31,32]. It is of great significance to develop novel biomaterials that replicate diverse cellular microenvironments and meet the varied requirements of tissue engineering. Amino acid-based poly(ester amide) (PEA) could be a promising polymer for 3D printing scaffolds with good mechanical properties, elasticity, thermostability and biocompatibility, due to the incorporation of biodegradable polyesters, robust polyamides, and bioactive amino acid into one structure [[33], [34], [35]]. Gloria et al. showed the higher Young's modulus and improved cell adhesion, spreading and proliferation rates of PEA/PCL blend scaffolds than PCL scaffolds alone, indicating the superior mechanical properties and bioactivity of PEA [36]. COPMA is a collagen-derived biomaterial with good biocompatibility, rapid UV-curing, tunable mechanical and self-healing properties [37]. COPMA hydrogels could be a promising alternative for collagen and methacrylated gelatin hydrogels for tissue engineering, due to the good hydrophilicity and accessibility. Therefore, the combination of PEA scaffold and COPMA-MNP hydrogel with 3D cellular anisotropic architecture may be a promising hybrid construct for replicating *in vitro* mechanical robust anisotropic microenvironments, but has not been explored yet.

**Fig. 1:**
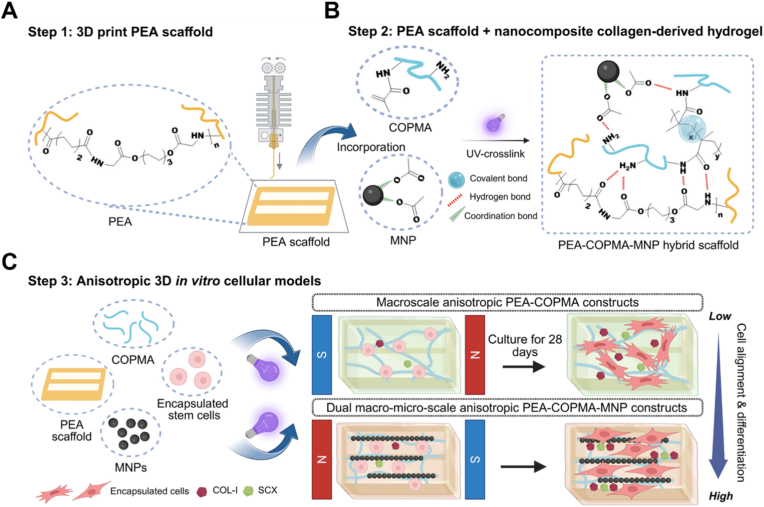
Overview of anisotropic soft-hard hybrid constructs. A) Synthesis and 3D printing of PEA scaffolds. B) Combination of 3D printed PEA scaffolds and nanocomposite collagen-derived hydrogels. C) Biofabrication of PEA-COPMA constructs with macroscale anisotropy and PEA-COPMA-MNP constructs with dual macro-micro-scale anisotropy, leading to differences in cell alignment and differentiation.

In this study, we develop novel oriented soft-hard hybrid constructs by integrating 3D printed elastic PEA scaffolds with anisotropic hydrogels, showing enhanced mechanical properties, 3D anisotropic structures and outstanding bioactivities. First, amino acid-based PEA is 3D printed into various organized architectures via FDM to provide mechanical support and macroscale anisotropy (Fig. 1A). Next, soft-hard hybrid scaffolds were prepared with good stability and elasticity by the formation of covalent and non-covalent bonds (Fig. 1B). Two biocompatible hybrid constructs, PEA-COPMA with macroscale anisotropy and PEA-COPMA-MNP with both macroscale and microscale anisotropy, were fabricated under simultaneous and rapid exposure to light and magnetic field (Fig. 1C). In the presence of dual-scale anisotropy, encapsulated hMSCs highly aligned along both the macroscale uniaxial PEA scaffold and the microscale anisotropic COPMA-MNP matrix, but disorganized in COPMA matrix alone without microscale anisotropy. Furthermore, hMSCs differentiation was further promoted in dual-scale anisotropic nanocomposite constructs, owing to the contribution of combined intrinsic biochemical cues with microscale biophysical anisotropic cues.

## 2 Materials and methods

### 2.1 Reagents

Glycine (99%), 1,6-hexanediol (97%), *p*-toluenesulfonic acid monohydrate (99%), triethylamine (99%), adipoyl chloride (98%), anhydrous dimethyl sulfoxide (DMSO, 99.9%), and acetic anhydride (99%) were purchased from Sigma-Aldrich. Calcium hydride (93%) and p-nitrophenol (99%) were purchased from ACROS Organics. Anhydrous calcium chloride (96%) was ordered from VWR. Toluene (99.8%), tert-butyl methyl ether (99.9%), ethyl acetate (99.5%), methanol (99.9%), acetone (99.8%), and acetonitrile (99.9%), were purchased from Biosolve. Deuterated DMSO (DMSO-*d*_6_, 99.9%) was purchased from BUCHEM BV. Other reagents were purchased from Sigma-Aldrich and used as received unless otherwise specified in the below sections.

### 2.2 Synthesis and characterization of PEA

The synthesis of monomers is reported in the supporting information. PEA was synthesized with an optimized version of our previous protocol [38]. Briefly, monomer A (di-p-toluenesulfonic acid salt of bis(glycine)hexane 1,6-diester; Gly-E, 71.10 mmol, 1.015 equiv) and monomer B (di-p-nitrophenyl adipate; NA, 70.05 mmol, 1 equiv) were dissolved in anhydrous DMSO (117.62 mL) under vigorous mechanical stirring at 60 °C with a nitrogen inlet. Triethylamine (Et_3_N; 156.42 mmol, 2.2 equiv) was freshly dried through refluxing over CaH_2_ to obtain anhydrous Et_3_N and then added dropwise to the mixture, which was reacted at 60 °C with continuous stirring for 2 h. This mixture solution was diluted by 183.38 ml of anhydrous DMSO, and then pre-endcapped by addition of Gly-E (1.42 mmol) and anhydrous Et_3_N (3.13 mmol) with stirring at 60 °C for another 2 h. Finally, end-capping was conducted by adding acetic anhydride (284.39 mmol) and Et_3_N (46.92 mmol) to the reaction mixture, followed by stirring at 60 °C for 1 h and subsequently at 40 °C for 2 h. The end-capped PEA solution was precipitated twice each in ethyl acetate and water, respectively. The purified PEA was vacuum dried at 50 °C for 24 h and 80 °C for 24 h to get yellowish powders with a yield of 62%. The characterizations of PEA are mentioned in the supporting information.

### 2.3 3D printing PEA scaffolds

For the formation of 3D scaffolds, several geometries were coded in the Rhinoceros 7.0 software with Grasshopper plugin (Version 7.37, Robert McNeel & Associates). Geometries were designed with morphological patterns varying between linear (6∗2.5∗1.0 mm, length∗width∗height), rectangular (10∗4∗1.0 mm, length∗width∗height), waved (10∗4∗1.0 mm, length∗width∗height), and circular (8∗8 mm, diameter∗height), and were translated to G-code for 3D printing. The PEA scaffolds were 3D printed using a Bioscaffolder (SysENG, Germany) at 185 °C and 5 bar through a G22 nozzle (inner diameter: 406 μm, DL Technology, USA) with a preflow duration of 0.4 s. To ensure homogeneous scaffold fabrication and smooth printing, parameters such as printhead speed (300-400 mm/min) and screw rotation speed (100-3000 R/min) were systematically optimized. The printed PEA scaffolds were imaged by a stereomicroscope (Nikon SMZ25, Japan).

### 2.4 Preparation of COPMA and COPMA-MNP hydrogels

*COPMA synthesis.* COPMA was prepared following our previously reported protocol [37], in which approximately 94% of the primary amines on collagen peptides were converted to methacrylamides (molecular weight approximately 3 kg/mol)

*MNPs synthesis.* MNPs were synthesized according to the procedure reported by Chen et al. [38]. Briefly, ferric chloride hexahydrate (1.8 g) and sodium acetate (2.04 g) were dissolved in ethylene glycol (70 mL) and stirred vigorously for 30 min. This mixture solution was then hydrothermally reacted at 200 °C for 12 h. After cooling to room temperature, MNPs were collected using a magnet and washed 3 times each with water and ethanol, respectively. The purified MNPs were stored in ethanol at 4 °C until further use. The morphology and size of MNPs were analyzed using transmission electron microscopy (TEM, FEI electron microscope, USA) and dynamic light scattering (DLS; Malvern Zetasizer Nano, Panalytical, UK). The crystalline composition of MNP was analyzed by X-ray diffraction (XRD, D2 phaser, Bruker, USA) using Cu Kα radiation (λ = 1.5406 Å).

*Synthesis of hydrogels.* A COPMA stock solution (30% w/v) was prepared in modified Dulbecco's phosphate-buffered saline (DPBS, without calcium or magnesium) at room temperature and sterilized by filtering through a 0.22 μm polyethersulfone (PES) syringe filter. This solution was diluted with DPBS to obtain 15% (w/v) COPMA. A photoinitiator stock solution (lithium phenyl-2,4,6-trimethyl-benzoyl phosphinate, LAP, 4% w/v) was prepared in DPBS and sterilized by filtration through a 0.22 μm PES filter. The sterilized COPMA solution with 0.1% LAP was mixed with or without MNPs to obtain hydrogel precursors at the final concentration of 15% (w/v) COPMA with/without 0.5% (w/v) MNPs. These hydrogel precursors were crosslinked under UV light (365 nm, 20 mW cm^−2^) for 3 min in the presence of a magnetic field (35 ± 2 mT).

### 2.5 Preparation of soft-hard hybrid scaffolds

The above COPMA or COPMA-MNP hydrogel precursors were injected to PEA scaffolds and then crosslinked under UV light (365 nm, 20 mW cm^−2^; UVP CL-1000 Ultraviolet Crosslinkers, Analytik Jena, Germany) for 3 min in presence of a magnetic field (35 ± 2 mT) created between 2 neodymium block magnets (46∗30∗10 mm, length∗width∗height, nickel-plated, N40, Supermagnete, Germany).

### 2.6 Mechanical tests of hydrogels and scaffolds

*In situ rheological tests.* Photorheological measurements were conducted using a DHR-2 rheometer (TA Instruments, USA) equipped with a cone-plate geometry (20 mm diameter, 2.002°, 53 μm gap). Oscillatory time sweeps were performed at 2% strain and frequency of 10 rad s^−1^, beginning without UV exposure for 1 min, followed by 4 min of UV irradiation (365 nm, 20 mW cm^−2^; M365LP1 LED, with DC2200 LED Driver modulation, Thorlabs, Germany). Oscillation frequency sweeps were then carried out from frequency of 1 to 100 rad s^−1^ at a constant strain of 2%. Finally, oscillatory strain amplitude sweeps were performed over a range of 0.1-10% strain at frequency of 10 rad s^−1^.

*Self-healing tests.* Strain sweep measurements were conducted over a range of 0.1-500% strain. Cyclic strain tests were performed by alternately applying 1% and 500% strain at room temperature. In addition, the hydrogel was cut and allowed to be rejoined at room temperature for 3 h.

*Tensile tests.* The PEA and PEA-COPMA-based scaffolds were subjected to 10% cyclic strain tests and tensile-to-failure tests at a rate of 0.2 mm/s using a TA ElectroForce system (TA Instruments, USA) equipped with a 450 N load cell. The height and diameter of each sample were measured with a caliper before each test.

### 2.7 Cell culture media and cell culture

The proliferation medium consisted of α-minimum essential medium (α-MEM) supplemented with 10% (v/v) fetal bovine serum (FBS), 1% (v/v) ascorbic acid, and 1% (v/v) penicillin-streptomycin (Pen-Strep, Thermo Fisher). The hMSCs (PromoCell, donor: 30-years-old Caucasian female) were cultured in T225 flasks at a seeding density of 1000 cells/cm^2^. Cells were passaged upon reaching 80% confluence, and experiments were performed using cells at passage 4.

### 2.8 hMSCs laden hybrid scaffolds

The hMSCs at a concentration of 4 × 10^6^ cells/mL were mixed with the hydrogel precursors and injected into PEA scaffolds. These constructs were immediately crosslinked in a 48-well plate under UV irradiation (365 nm, 20 mW cm^−2^; UVP CL-1000 Ultraviolet Crosslinkers, Analytik Jena, Germany) for 3 min in the presence of a magnetic field (35 ± 2 mT). Subsequently, crosslinked hybrid constructs were cultured at 37 °C in a 5% CO_2_ atmosphere without light and magnetic field. The culture medium was refreshed every 2 days.

### 2.9 Live/dead staining

The hybrid constructs were collected on days 2 and 28, rinsed with DPBS, and incubated in 2 μM calcein acetoxymethyl ester (Calcein AM, Thermo Fisher) for 30 min at 37 °C in the dark. Ethidium homodimer-1 solution (EthD-1, Thermo Fisher) was then added to achieve a final concentration of 0.06 μM, followed by a 25 min incubation at 37 °C. After rinsing twice with DPBS, the constructs were imaged using a fluorescence microscope (Eclipse Ti-E, Nikon, Japan).

### 2.10 Metabolic activity

Metabolic activity of hMSCs was assessed using Cell Titer-Glo 3D assay (Promega) on days 0, 2, 14 and 28. Equal volumes of reagent and culture medium were added to the hybrid constructs and mixing by pipetting. Following a 30-min incubation at room temperature, 100 μL of supernatant was transferred to a white-bottom 96-well plate, and luminescence was measured using a CLARIO star plate reader (BMG Labtech, Germany).

### 2.11 DNA quantification

Cells were extracted from hybrid constructs by performing three freeze-thaw cycles, followed by overnight digestion at 56 °C with 1 mg/mL proteinase K and an additional three freeze-thaw cycles. DNA quantification was conducted using the CyQuant cell proliferation assay kit (Thermo Fisher) according to the manufacturer's instructions.

Briefly, the extracted supernatant was incubated with lysis buffer containing RNase for 2 h at room temperature. Subsequently, an equal volume of 2× GR-dye solution was added, followed by a 15 min incubation. Samples (200 μL) were transferred to a black 96-well plate, and fluorescence was measured at 520 nm by a CLARIO star plate reader.

### 2.12 GAG quantification

Cell extracts (25 μL) were mixed with calcium chloride (5 μL) and 1,9-dimethylmethylene blue solution (150 μL) in a 96-well plate. Absorbance was measured at 525 nm and 595 nm using a CLARIO star plate reader, and the difference between these values was used for quantification.

### 2.13 Immunostaining of COL-I and SCX

Hybrid constructs were fixed in 4% paraformaldehyde (PFA) and then rinsed with PBS. Constructs were blocked and permeabilized overnight at 4 °C in PBS containing 1% (w/v) triton X-100, 0.05% (w/v) tween-20, 5% (w/v) goat serum, and 1% (w/v) bovine serum albumin (BSA, VWR). After removal of the above solution, constructs were incubated at 4 °C for 24 h with primary antibodies: mouse anti-collagen I (COL-I, 1:200, ab6308, Abcam) and rabbit anti-scleraxis (SCX, 1:200, ab58655, Abcam). Following three washes with washing buffer (1% BSA, 0.05% tween-20 in PBS), samples were incubated overnight at 4 °C with secondary antibodies (goat anti-mouse and goat anti-rabbit, 1:500 in washing buffer, Abcam). After washing, F-actin was stained with Alexa Fluor Phalloidin 568 (1:75, Thermo Fisher) for 1 h at room temperature, followed by nuclear staining with DAPI for 20 min in the dark. Hybrid constructs were rinsed with PBS and imaged using a confocal microscope (Leica TCS SP8 CARS, Germany). Quantifications of MNP chains and cell angle distribution, F-actin area, COL-I and SCX expression area were calculated using the General Analysis (GA3) module of NIS-Elements software (Nikon, version 5.30.3, Japan).

### 2.14 Statistical analysis

Statistical analyses were conducted using GraphPad Prism software (version 10.3, USA). Comparisons between groups were performed using Student's *t*-test, one-way ANOVA, or two-way ANOVA followed by Tukey's multiple comparison test. Data are expressed as mean ± standard deviation (SD), or with error bars representing SD in graphical representations. Statistical significance was defined as ∗*p* < 0.05; ∗∗*p* < 0.01; ∗∗∗*p* < 0.001 and ∗∗∗∗*p* < 0.0001.

## 3 Results

### 3.1 PEA synthesis and 3D printing PEA scaffolds

Amino acid-based PEA has been studied in various biomedical fields, such as drug delivery, vascular tissue engineering, bone regeneration, and wound healing [[39], [40], [41], [42]]. As biodegradable thermoplastic biomaterials, PEA has been endowed with different architectures via biofabrication techniques (e.g., electrospinning and fused deposition modelling) for tissue engineering [43,44]. However, the potential of uniaxial PEA scaffolds fabricated via 3D printing to direct stem cell alignment and differentiation remains unexplored. Thus, in this section, we synthesized glycine functionalized PEA and fabricated uniaxial PEA scaffolds via additive manufacturing.

**Fig. 2:**
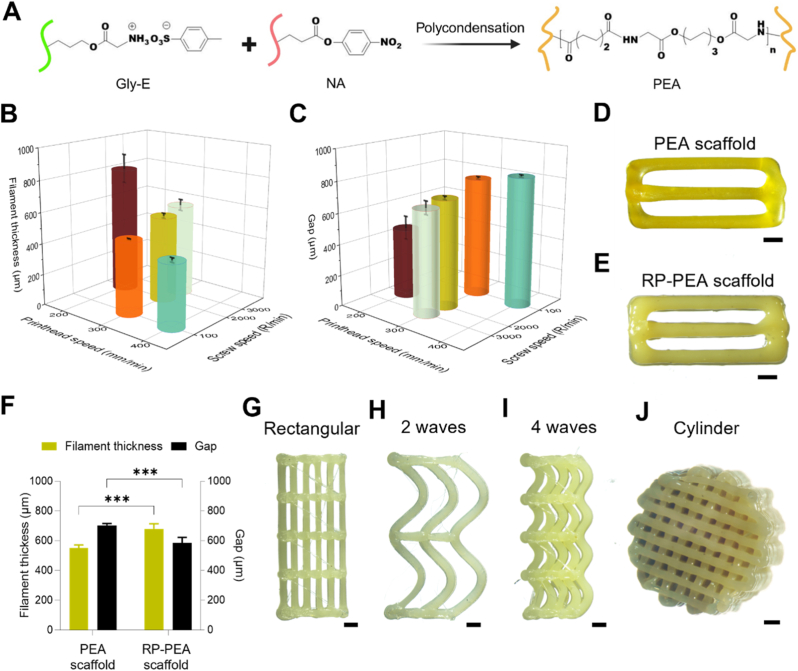
Synthesis of PEA and 3D printing PEA scaffolds. A) PEA synthesis via solution polycondensation reaction. B-C) Comparison of effects of printhead speed and screw speed on the filament thickness and gap in PEA scaffolds. D-E) Optical images of PEA scaffolds and RP-PEA scaffolds, scale bar: 1000 μm. F) Comparison of effects of printhead speed and screw speed on the filament thickness and gap in the optimized PEA scaffolds and RP-PEA scaffolds (n = 3, ∗∗∗*p* < 0.001). G-J) PEA scaffolds in different geometries, scale bar: 1000 μm.

PEA was synthesized efficiently by solution polycondensation of di-p-toluenesulfonic acid salt of bis(glycine)hexane 1,6-diester (Gly-E) and di-p-nitrophenyl adipate (NA; Fig. S1) under mild conditions (60 °C for 2 h; Fig. 2A, and Fig. S2). The synthesized PEA exhibited a moderate number average molecular weight (Mn = 44.6 kg/mol) and a narrow dispersity (Ð = 2.3; Table S1). PEA showed the glass transition temperature (T_g_) at 22 °C, indicating a solid state at room temperature (Table S1). PEA showed 5% weight loss temperature (T_5%_) at 342 °C and melting temperature (T_m_) at 167 °C (Table S1 and Fig. S3). These findings indicate their good thermostability and possibility for additive manufacturing into organized morphology above the melting temperature.

To enable smooth and continuous 3D printing of PEA into homogeneous scaffolds, printing parameters, including printhead speed and screw speed, were optimized (Table S2 and Fig. S4). Specifically, at a screw speed of 100 R/min, increasing the printhead speed endowed scaffolds with significantly thinner filaments and larger inter-filament gaps (Fig. S4A-B and Fig. 2B-C, orange and green columns). At a printhead speed of 300 mm/min, higher screw speeds resulted in thicker filaments and smaller inter-filament gaps (Fig. S4B-D, Fig. 2B-C, orange, yellow and beige columns). Printing smoothness and scaffold homogeneity were improved at a screw speed of 2000 R/min compared with 3000 R/min, with a printhead speed of 300 mm/min (Fig. S4C-D, Fig. 2B-C, yellow and beige columns). Under a screw speed of 2000 R/min, increasing the printhead speed led to a significant reduction in filament thickness and an increase in inter-filament gap (Fig. S4C, S4E, and Fig. 2B-C, brown and yellow columns). Overall, based on these results, the optimized printing parameters for PEA were 185 °C, 5 bar, a printhead speed of 300 mm/min, and a screw speed of 2000 R/min. According to these optimized parameters, PEA was smoothly 3D printed into an organized PEA scaffold with longitudinally aligned architecture (Fig. 2D).

Although PEA is a thermoplastic polymer, reports on its recycling via additive manufacturing are limited. Herein, we simply recycled the printed PEA scaffolds and reprinted them into organized scaffolds again under the same optimized printing parameters (Fig. 2E). Compared with the initial print, the recycled-printed PEA (RP-PEA) scaffolds showed higher filament thickness and reduced inter-filament gaps (Fig. 2F). This may be due to the lower M_n_ (36.4 kg/mol) after additive manufacturing at high temperature when compared to the unprinted PEA, resulting in decreased viscosity of PEA. This trend was consistent with the findings of Ansari et al. [45]. Notably, semicrystalline PEA could be 3D printed into diverse architectures, including linear, rectangular, wavy, and cylindrical geometries (Fig. 2G–J), enabling the fabrication of customized scaffolds tailored to specific defect shapes for tissue engineering applications. These 3D printed scaffolds showed high similarity to the computer-aided designed (CAD) models in terms of morphology, length, width and height (Fig. S5).

### 3.2 Dynamic mechanical properties of PEA-COPMA-based hybrid scaffolds

We have developed uniaxial PEA scaffolds for inducing macroscale anisotropy. Next, we have incorporated magnetic responsive MNPs into COPMA hydrogel networks to develop microscale anisotropic COPMA-MNP hydrogels. To further develop elastic and hierarchical hybrid scaffolds, we UV crosslinked microscale COPMA-MNP hydrogels in uniaxial PEA scaffolds under the exposure of magnetic field for 3 min.

MNPs were prepared using a hydrothermal method published previously [38]. TEM images showed that the synthesized MNPs had a round morphology and size distribution of 337.8 ± 40.6 nm (Fig. S6A–B). Moreover, DLS confirmed the high homogeneity of MNPs with hydrodynamic size of 1318.7 ± 83.5 nm and a low polydispersity index value (0.116 ± 0.054) (Fig. S6C). In addition, XRD analysis showed prominent diffraction peaks at 2θ values of 30.3°, 35.6°, 43.3°, 53.8°, and 57.3° (Fig. S6D), corresponding to (220), (311), (400), (422), and (511) crystallographic planes of magnetite Fe_3_O_4_ (JCPDS card No. 19-0629), respectively [46]. Notably, no diffraction peak corresponding to the (104) plane of Fe_2_O_3_ was observed at 2θ = 33.15° (JCPDS card No. 89-0598), confirming the absence of Fe_2_O_3_ impurities and indicating that the synthesized MNPs consisted solely of Fe_3_O_4_ [47].

**Fig. 3:**
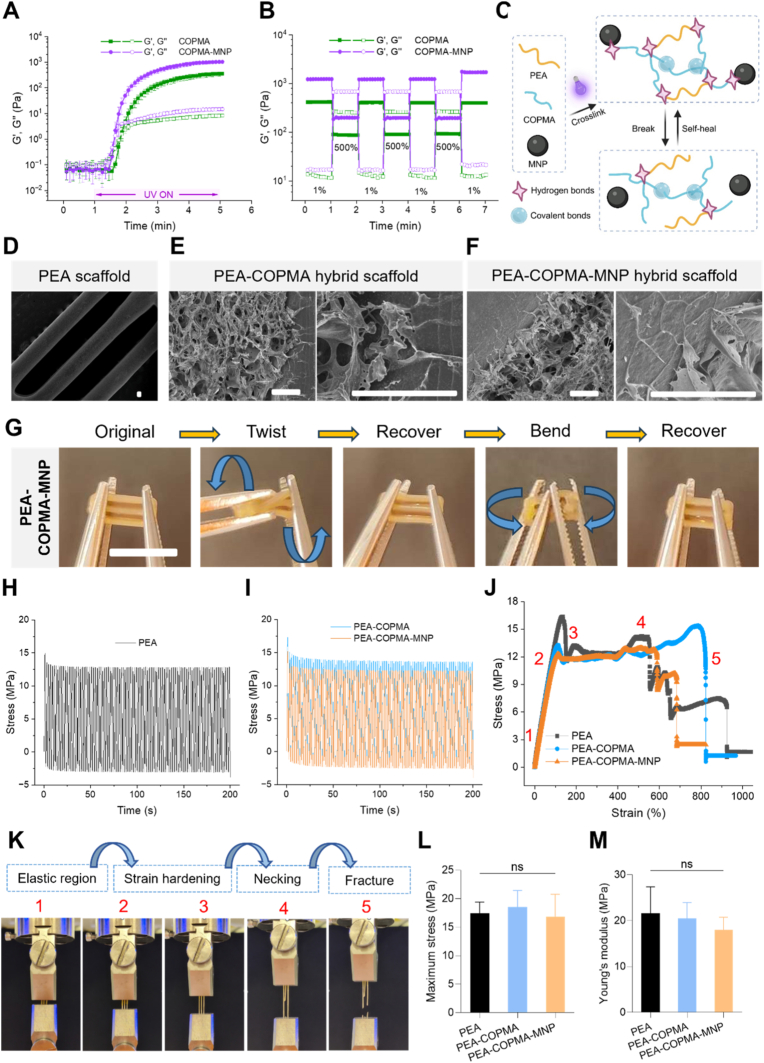
Soft-hard scaffolds with softness, toughness and elasticity. A) Photorheology of COPMA and COPMA-MNP hydrogels. B) Cyclic strain tests of COPMA and COPMA-MNP hydrogels at 1% and 500% strains. C) Illustration of covalent and non-covalent bonds in hybrid scaffolds. D-F) SEM images of PEA scaffolds, PEA-COPMA and PEA-COPMA-MNP hybrid scaffolds, scale bar: 100 μm. G) Elasticity of soft-hard hybrid scaffolds, scale bar: 6 mm. H-I) Cyclic tensile tests of PEA scaffolds, PEA-COPMA and PEA-COPMA-MNP hybrid scaffolds at 10% strains for 100 cycles. J) Tensile stress-strain curves of PEA scaffolds, PEA-COPMA and PEA-COPMA-MNP hybrid scaffolds. K) Representative images showing the five stages of the tensile testing process for PEA scaffolds. L-M) Maximum tensile stress and Young's modulus of PEA scaffolds, PEA-COPMA and PEA-COPMA-MNP hybrid scaffolds (n ≥ 3, ns indicates no significant difference).

COPMA and COPMA-MNP were developed as hydrogel sources and their *in situ* photocrosslinking behaviors were evaluated within the linear viscoelastic region on a rheometer (Fig. S7A–B). As shown in the photorheological curves, the storage modulus of COPMA-based hydrogels increased rapidly within 3 min, indicating their readily UV-crosslinking properties (Fig. 3A). In COPMA-MNP hydrogels, MNPs incorporated as physical fillers reinforced the hydrogel network, resulting in a higher storage modulus (1033 ± 107 Pa) than COPMA alone (355 ± 62 Pa, Fig. 3A). This indicated that non-covalent interactions primarily contributed to the mechanical properties of hydrogels. Given the abundance of non-covalent bonds in COPMA and on the surface of MNPs, we further evaluated the self-healing properties of COPMA-based hydrogels by performing strain sweep and cyclic strain tests across a strain range of 1% to 500%. Hydrogel network rupture occurred at approximately 325% strain for COPMA and 191% strain for COPMA-MNP (Fig. S7C). Notably, both COPMA-based hydrogels demonstrated self-healing capability across the 1–500% strain range, suggesting their potential to adapt to tissue deformation under dynamic physiological conditions (Fig. 3B). Besides, two pieces of hydrogels could be rejoined, which could sustain gravity and a certain strength without interfacial separation (Fig. S8). This self-healing property is likely attributed to the dynamic non-covalent interactions between COPMA and MNPs (Fig. 3C). These findings were consistent with some reported self-healable hydrogels with both covalent and non-covalent bonds [[48], [49], [50]].

Consequently, hybrid scaffolds were fabricated by integrating COPMA-based hydrogels with PEA scaffolds through a straightforward process—pouring hydrogel precursors into the gap of PEA scaffolds. Subsequently, mild UV-light (365 nm, 20 mW cm^−2^) and a weak magnetic field (35 ± 2 mT) were simultaneously applied for 3 min. Compared to the PEA scaffold itself (Fig. 3D), the PEA-COPMA (Fig. 3E) and PEA-COPMA-MNP hybrid scaffolds (Fig. 3F) exhibited porous structures between the scaffold filaments. Notably, both PEA scaffolds and hybrid soft-hard scaffolds could recover their original shape after being twisted and bent, demonstrating excellent elasticity (Fig. 3G, Fig. S9). Although undergoing twisting and bending, these hydrogels remained attached to the scaffolds to maintain integration (Fig. 3G), likely due to the dynamic hydrogen bonds between PEA and COPMA (Fig. 3C).

The elasticity and mechanical performance of PEA scaffolds, PEA-COPMA, and PEA-COPMA-MNP hybrid scaffolds were further quantified through tensile testing. Fatigue tests were first conducted at 10% strain to mimic the physiological strain experienced by connective tissues during daily activities. As shown in Fig. 3H, the PEA scaffolds could sustain 10% strain even for 100 cycles at 0.5 Hz. This good elasticity was also observed in the hybrid scaffolds (Fig. 3I). The stress-strain curves confirmed high elasticity in all these scaffolds, with yield strains exceeding 400% (Fig. 3J). Interestingly, these scaffolds exhibited five distinct elongation stages until failure (Fig. 3J–K). In stages 1-2 (linear elastic region), scaffold filaments elongated under stretching. Continued extension during stages 2-3 (strain hardening stage) and 3-4 (necking stage) further elongated the fibers until the maximum length was reached. In stage 5 (fracture stage), complete filament rupture occurred, resulting in tensile failure (Fig. 3K). Quantitatively, the maximum tensile stress was 17.5 ± 1.7 MPa for PEA scaffolds, 18.5 ± 2.4 MPa for PEA-COPMA scaffolds, and 16.9 ± 3.2 MPa for PEA-COPMA-MNP scaffolds, without statistically significant differences (Fig. 3L). All these scaffolds showed high Young's modulus at approximately 20 MPa, without significant differences between the PEA scaffolds and soft-hard hybrid scaffolds (Fig. 3M). However, the tensile strengths of COPMA-based hydrogels could not be obtained because these hydrogels were too brittle to be clamped properly on the mechanical tester. Overall, the comparison between the low rheological strength of hydrogels alone (storage modulus ∼1 kPa) and the high tensile strength of PEA scaffolds and soft-hard hybrid scaffolds (Young's modulus ∼20 MPa) indicated that PEA scaffolds endowed soft-hard hybrid scaffolds with good elasticity and resilience. Moreover, the nanocomposite COPMA-based hydrogels provided hydrous soft matrices with microscale anisotropy, which may facilitate cell spreading.

To compare the mechanical properties of PEA scaffolds and recycled-printed PEA (RP-PEA) scaffolds, the cyclic tensile test and failure tensile test were conducted on RP-PEA scaffolds as well. Similar to PEA scaffolds, RP-PEA scaffolds could also sustain 10% strain for 100 cycles (Fig. S10A). However, compared with PEA scaffolds, RP-PEA scaffolds displayed reduced elasticity, characterized by the absence of extended elongation stages and a lower yield strain (<200%) (Fig. S10B). Interestingly, RP-PEA scaffolds exhibited a higher maximum tensile stress (23.4 ± 1.2 MPa; Fig. S10C) but a comparable Young's modulus (17.8 ± 1.0 MPa; Fig. S10D) when compared to PEA scaffolds, indicating the possibility of recycling PEA scaffolds.

### 3.3 Magnetic response and biocompatibility in PEA-COPMA-based constructs

**Fig. 4:**
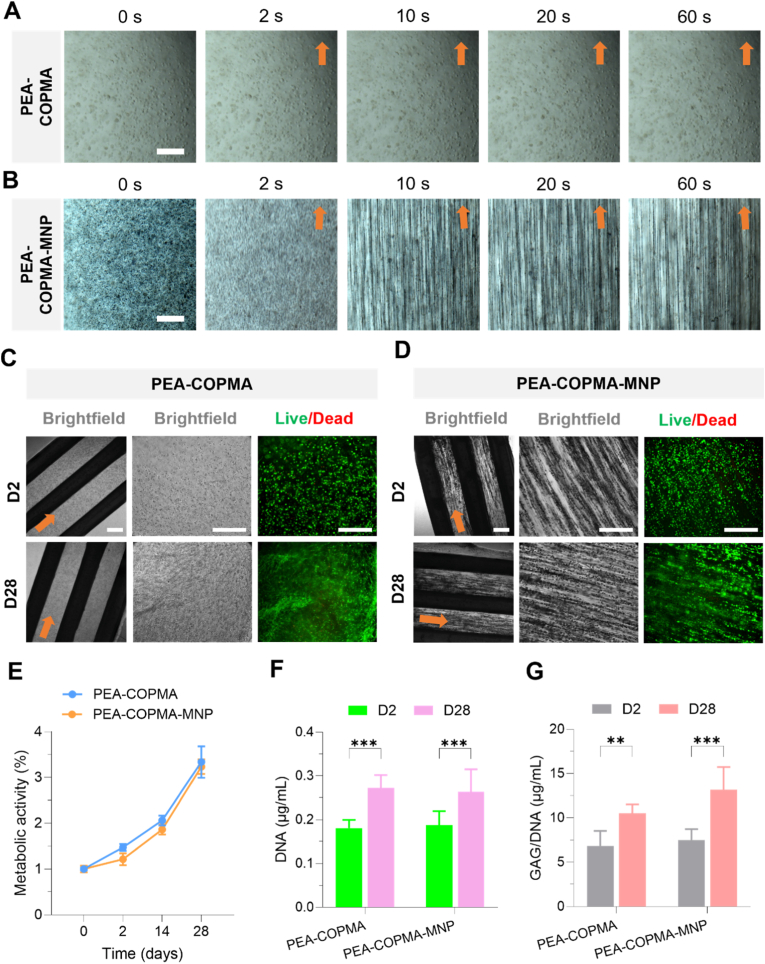
Biocompatibility and GAG production of hMSCs laden PEA-COPMA-based hybrid scaffolds up to 28 days. A) Encapsulated hMSCs showed no orientation in COPMA hydrogel precursors after exposure to magnetic fields. B) Encapsulated hMSCs showed rapid magnetic-responsive orientation in COPMA-MNP hydrogel precursors in 10 s under magnetic fields. Scale bar: 200 μm. Orange arrows indicate the magnetic field direction. C-D) Live/dead staining of hybrid constructs on day 2 and 28, scale bar: 200 μm. Orange arrows indicate the magnetic field direction during the gelation process. E) Metabolic activity of encapsulated hMSCs in hybrid constructs. F) DNA content in constructs. G) Normalized GAG production in constructs (n ≥ 3, ∗∗*p* < 0.01, ∗∗∗*p* < 0.001). (For interpretation of the references to colour in this figure legend, the reader is referred to the Web version of this article.)

Given the inherent anisotropic architecture of ligaments/tendons, matrices with anisotropic topography play a crucial role in directing cell growth, ECM deposition, and tissue regeneration [51]. To fabricate anisotropic cell-laden hybrid scaffolds, we employed a straightforward approach by parallelly placing two commercial magnets on both sides of the cell-laden scaffold-hydrogel precursors during UV irradiation. Despite exposure to the magnetic field (35 ± 2 mT), cells were distributed randomly in the constructs without MNPs (Fig. 4A). In contrast, in the PEA-COPMA-MNP constructs, MNPs assembled into oriented chains in 10 s and stabilized from 20 to 60 s, aligning in the direction of the magnetic field (Fig. 4B). Interestingly, the hMSCs moved along and were compressed in between the gaps of oriented MNP chains (Fig. 4B). These findings indicated that MNPs in hMSCs-laden hydrogels showed rapid magnetic response, which may benefit cell alignment during cell culture. Because of its rapid light and magnetic response, we fabricated cell-laden soft-hard hybrid scaffolds by exposing them to magnetic field (35 ± 2 mT) and UV-light (365 nm, 20 mW cm^−2^) simultaneously during a 3-min gelation process, but neither light nor magnetic fields were applied during cell culture.

The biocompatibility of hMSCs laden PEA-COPMA-based hybrid scaffolds was investigated by live/dead staining, metabolic activity and DNA quantification. Live/dead images revealed high cell viability in all constructs with magnetic guidance, indicating these constructs did not impair cell survival (Fig. 4C–D). Notably, hMSCs in PEA-COPMA-MNP constructs aligned parallel to the anisotropic guidance (Fig. 4D), whereas those in PEA-COPMA constructs remained randomly spread despite exposure to the same magnetic field (Fig. 4C). The metabolic activity of encapsulated hMSCs increased more than 3-fold in all hybrid constructs over 28 days of culture (Fig. 4E), and the DNA content also increased over time in these constructs (Fig. 4F). Glycosaminoglycans (GAGs), one of the most abundant ECM components in connective tissues, were quantified as a marker of ECM production in the constructs [52]. Both hybrid scaffolds exhibited a significant increase in GAG production over 28 days (Fig. S11). Moreover, PEA-COPMA-MNP constructs showed significantly higher GAG/DNA levels than PEA-COPMA constructs, suggesting enhanced ECM production and stem cell differentiation under the biophysical anisotropic cues (Fig. 4G). Collectively, these results indicated the good biocompatibility of the hybrid scaffolds with or without magnetic nanoparticles during long-term culture. The higher DNA and GAG/DNA contents observed in MNP-laden anisotropic constructs may be associated with anisotropic mechanotransduction induced by the combined macro-/micro-scale anisotropic microenvironment. This anisotropic microenvironment may activate key mechanotransduction pathways involving Piezo1 and/or YAP/TAZ, and ultimately facilitate matrix production [[53], [54], [55]].

### 3.4 Stability of anisotropic hybrid constructs

**Fig. 5:**
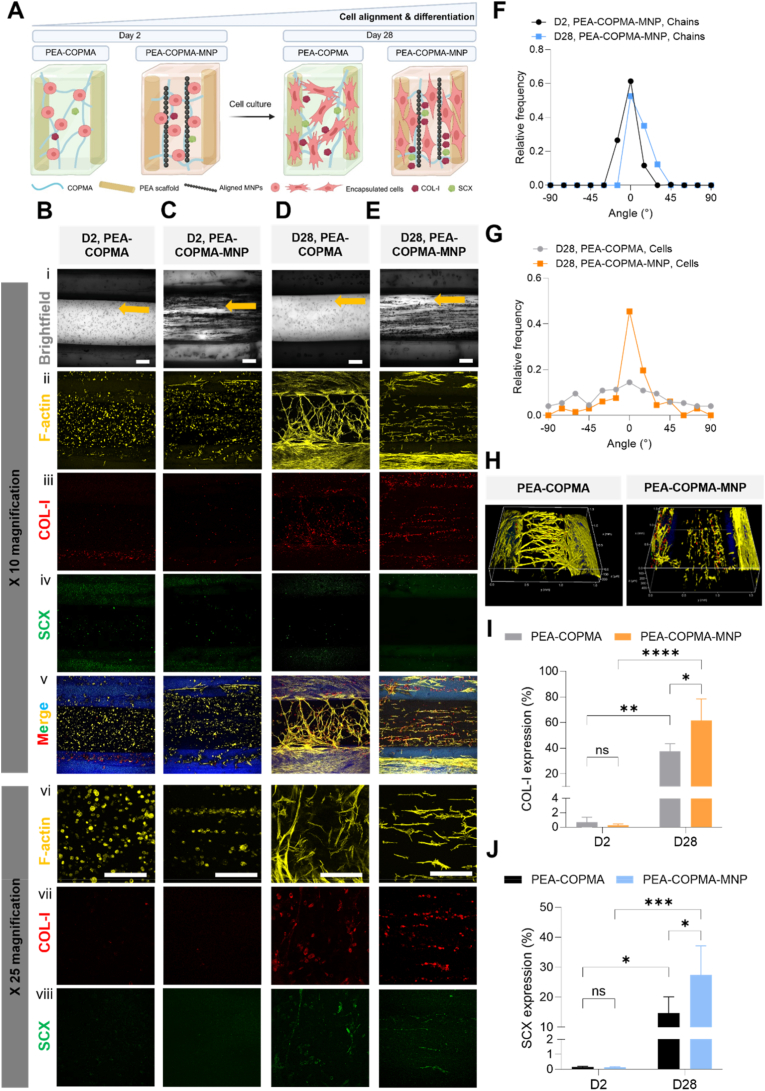
Comparison of cell alignment and differentiation in soft-hard hybrid constructs. A) Illustration of hybrid constructs with or without biophysical anisotropic cues on days 2 and 28. B-E) Confocal images of hybrid constructs on day 2 (B-C) and on day 28 (D-E), scale bar: 100 μm. Orange arrows indicate the direction of the magnetic field. F) Stability of MNPs chains in constructs during 28-day culture. G) Angle distribution of cells in constructs. H) 3D reconstruction of PEA-COPMA and PEA-COPMA-MNP constructs on day 28. I-J) COL-I and SCX expression area percentage (n ≥ 3, ns indicates no significant difference, ∗*p* < 0.05, ∗∗*p* < 0.01, ∗∗∗*p* < 0.001, ∗∗∗∗*p* < 0.0001). (For interpretation of the references to colour in this figure legend, the reader is referred to the Web version of this article.)

To investigate the effects of biophysical anisotropic cues and intrinsic biochemical cues on 3D cell alignment and differentiation, hMSCs were encapsulated in the PEA-COPMA and PEA-COPMA-MNP hybrid scaffolds (Fig. 5A). First, the stability of anisotropic topography of cell-laden scaffolds was investigated. In the PEA-COPMA constructs, only PEA filaments showed uniaxial morphology, whereas the COPMA hydrogels displayed no anisotropic features, indicating that PEA-COPMA constructs only possessed macroscale anisotropy (Fig. 5Bi, 5Di). In contrast, PEA-COPMA-MNP constructs showed both uniaxial PEA filaments and distinctly aligned MNP chains as early as day 2, which remained over 28 days (Fig. 5Ci, 5Ei), indicating that PEA-COPMA-MNP constructs possessed both macroscale and microscale anisotropy. Quantitative angle distribution analysis revealed no significant change in MNP chain alignment over 28 days, indicating their high structural stability during cell culture (Fig. 5F). These findings indicated that the simple and rapid exposure of constructs to a weak magnetic field (3 min, 35 ± 2 mT) could efficiently fabricate magnetically induced anisotropic constructs, and cell-matrix interactions did not compromise the excellent stability of magnetically responsive anisotropic topography.

### 3.5 Biophysical topography regulates 3D cell alignment

Subsequently, the effect of biophysical anisotropic cues on 3D cell organization was evaluated. On day 2, hMSCs dispersed homogenously in both hybrid constructs (Fig. 5B and Cii). In the PEA-COPMA construct without hydrogel microscale alignment, hMSCs spread along the PEA scaffolds but elongated randomly within COPMA bridging the gaps between adjacent PEA filaments during 28 days of culture, indicating outstanding cell-material interactions (Fig. 5Dii). In PEA-COPMA-MNP constructs with dual macro-micro-scale alignment, the encapsulated hMSCs spread aligned in both PEA scaffolds and COPMA-MNP hydrogels on day 28, indicating the efficacy of anisotropic cues in directing cell alignment (Fig. 5Eii). Quantitatively, the cell dispersity angle in PEA-COPMA constructs was broadly distributed (Fig. 5G, grey symbol), but significantly concentrated at approximately 0° in PEA-COPMA-MNP constructs (Fig. 5G, orange symbol). After 28 days of incubation, a well-aligned 3D cell network was observed throughout the whole PEA-COPMA-MNP constructs (Fig. 5H, right). However, in PEA-COPMA constructs, cell alignment was only shown in PEA filaments, but not in the COPMA matrix (Fig. 5H, left). These aligned cell networks are fundamental for modeling the anisotropic cellular environment to further investigate the effect of biophysical anisotropic cues on cell-matrix interactions and stem cell differentiation.

### 3.6 Biophysical anisotropic cues and inherent biochemical cues regulate hMSCs differentiation

Furthermore, the influence of biophysical anisotropic cues and intrinsic biochemical cues on cell differentiation was assessed by the expression of collagen type I (COL-I) and scleraxis (SCX), two representative markers of ligamentogenic/tenogenic differentiation, as an example to indicate stem cell differentiation capacity in hybrid constructs [56]. Herein, we did not introduce any exogenous biochemical cues (e.g., growth factors) in the constructs/culture system. We investigated whether collagen-derived matrices could act as intrinsic biochemical cues to stimulate hMSCs differentiation and investigated their combined effect with biophysical anisotropic cues on cell differentiation. Immunostaining images (Fig. 5B-Cvii-viii) and quantitative analysis of biomarker expressions (Fig. 5I–J, left columns) revealed a low amount of COL-I and SCX in both constructs on day 2. After 28 days of incubation, COL-I expression was significantly increased in both constructs (Fig. 5D-Eiii, vii). However, due to the strong autofluorescence of PEA scaffolds in the green and blue channels (Fig. 5B–Eiv-v), SCX and nuclear signals could not be quantified across the entire hybrid constructs. Thus, COL-I and SCX expression levels were quantified exclusively within the hydrogel regions at 25× magnification to eliminate PEA-related autofluorescence (Fig. 5B–Evi-viii). Although without the addition of any exogenous bioactive growth factors, both constructs showed significantly higher COL-I and SCX expression at day 28 compared to day 2, indicating stem cell differentiation toward ligamentocytes/tenocytes (Fig. 5I–J, left columns vs. right columns). PEA-COPMA constructs promoted cell differentiation, likely due to its abundant collagen peptide environment that mimicked the collagen-rich ECM and facilitated collagen deposition for cell differentiation, consistent with our previous and another existing studies [37,57]. Furthermore, aligned PEA-COPMA-MNP constructs incorporating both intrinsic biochemical cues and biophysical anisotropic cues showed significantly more COL-I and SCX expressions on day 28 compared to constructs lacking anisotropic MNPs (Fig. 5I–J, right columns). These findings indicated the complementary effect of intrinsic biochemical signals and biophysical anisotropic guidance in driving cell differentiation within biomimetic constructs that replicated anisotropic cellular microenvironments by mimicking its collagen-rich component and 3D anisotropic cellular structure.

## 4 Discussion

The development of mechanically reinforced, anisotropic ECM-mimicking models is critical for elucidating cell-ECM interactions *in vitro* and guiding the rational design of biomimetic constructs for tissue engineering [58]. Cell-laden natural hydrogels are widely employed as artificial 3D ECMs, but typically exhibit low tensile strength and isotropic architecture, limiting their biomimetic performance [59,60]. In contrast, thermoplastic scaffolds often show high stiffness and can be fabricated into organized shapes, but do not replicate hydrous 3D cellular matrices *in situ* [61]. Combining naturally derived hydrogels with synthetic polymer-based stiff scaffolds may offer a promising strategy to integrate bioactivity, biocompatibility, durability, and mechanical reinforcement in a hybrid construct. In this work, we developed 3D hybrid soft-hard constructs by integrating naturally derived microscale anisotropic nanocomposite hydrogels with a macroscale anisotropic, elastic, and robust amino acid-based scaffold to modulate the alignment and differentiation of encapsulated stem cells.

PEA is a promising polymer for tissue engineering due to its good tunability, biodegradability and biocompatibility. Conventional FDM-printable polymers such as PCL and PLA have been widely reported to be used in absorbable sutures, orthopedic fixation systems, drug-delivery depots, and tissue-engineering scaffolds [62,63]. However, the degradation of PCL is significantly slow, which may limit tissue remodeling in regenerative applications [64,65]. PLA can produce acidic products after degradation that may induce local inflammatory responses [66,67]. In contrast, amino-acid-based PEAs show tunable biodegradability due to the presence of both hydrolytically degradable ester groups and more stable amide groups within the polymer backbone. Their tunable degradation is also associated with their hydrophilicity, crystallinity, amino-acid composition, and the presence of enzymes [68,69], which can be customized to adapt to support cell recruitment, ECM deposition and tissue regeneration, facilitating clinical applications. Tsitlanadze et al. showed that PEA films can be completely biodegraded in rats within 1-2 months with lipase-impregnation and 3-6 months without lipase-impregnation [70]. Besides, Ansari et al. showed that PEAs can be fabricated into mechanically stable scaffolds with organized architectures for tissue engineering [44]. However, the rheological and thermal processing behavior of PEAs remains comparatively underexplored. Our previous study showed that the printability of PEA scaffolds strongly depends on molecular weight and end-group of PEAs, printing temperature, melt viscosity, cooling behavior, and rheology [45]. Future improvements in PEA printability may be achieved through optimization of nozzle diameter, extrusion temperature, deposition speed, thermal control, and polymer molecular weight. Additionally, similar as PCL, PEA can be potentially functionalized with photocrosslinkable groups (e.g., methacrylamide) to enable light-based printing [71]. This would require substantial polymer redesign to achieve suitable photocuring kinetics, viscosity, and optical properties.

Hydrogels with an aligned architecture are essential for replicating anisotropy in *in vitro* tissue models. Various strategies have been developed to fabricate anisotropic hydrogels, but achieving an *in situ* 3D alignment cellular microenvironment in cell-laden hydrogels through a robust and simple preparation process remains a significant challenge [[72], [73], [74], [75]]. For example, Gu et al. synthesized concentric fibrous honeycomb hydrogels by concentric ice-templating, compression, freeze-drying and reswelling method [76]. Although this approach successfully produced an anisotropic architecture, the alignment process was not compatible with living cells. Recently, biomimetic anisotropic cell-laden hydrogels have been engineered by spatially controlling the orientation of magnetic responsive nano/microfillers [77,78]. For example, Teixeira et al. produced magnetically aligned MNPs-laden microfibers and incorporated them into gelatin hydrogels to induce alignment of human adipose tissue-derived stem/stromal cells [79]. However, this approach still required multiple complex steps, including mixing MNPs with PCL in organic solvents, electrospinning MNP-PCL fibers, and cryo-sectioning short magnetic microfibers. To simplify the process, several studies have attempted to induce anisotropic constructs by directly incorporating MNPs into cell-laden hydrogels [[80], [81], [82]]. While this straightforward method enables easy fabrication of oriented 3D hydrogels, maintaining a stable 3D anisotropic microenvironment for a long period (e.g., 28 days) remains challenging, likely due to limited cell attachment or weak cell-matrix interactions. However, in our study, we successfully achieved *in situ* 3D stem cell alignment and cell differentiation within an oriented cellular microenvironment for one month by directly incorporating the MNPs into COPMA hydrogels. This prolonged stability and functionality are likely attributed to the stable covalent and non-covalent networks in hydrogels and the complementary effect between biophysical anisotropic cues (aligned morphology in nanocomposite constructs) and biochemical cues (bioactive collagen-derived matrix).

Fabricating scaffold-hydrogel hybrid constructs with anisotropic cellular networks and reinforced mechanical properties offers a promising approach for replicating mechanically robust anisotropic ECM, but existing strategies require further improvement and simplification [83,84]. For example, Wei et al. incorporated aligned cryogel fibers (ACF) into 3D printed methacrylated gelatin (GelMA) scaffolds (PS) by unidirectional freeze casting techniques, and noticed the seeded cells gradually grew aligned within the PS/ACF [85]. Yao et al. assembled melt-electrowritten PCL tubular scaffolds consisting of 5-layer crimped fibers and collected fibers within a GelMA hydrogel by surface crystallization and rolling. The hybrid scaffolds obtained a strong tensile strength of 6.94 MPa [86]. Notably, our hybrid constructs indicated a more efficient and straightforward strategy to obtain soft-hard composites by directly UV-crosslinking the cell-laden hydrogel precursor between the PEA filaments under a mild magnetic field within 3 min. Despite this simple fabrication process, the PEA scaffold and COPMA-based hydrogel hybrid constructs exhibited excellent structural integrity, maintaining adhesion and shape even after twisting, bending, and elongation. These hybrid scaffolds presented complex anisotropic microenvironments and high Young's moduli of approximately 20 MPa under tensile loading. Combining high elasticity, robust tensile strength, and a 3D anisotropic cellular network, these hybrid constructs may hold strong potential for ligament/tendon tissue engineering.

Compared to other anisotropic soft-hard hybrid scaffolds, our anisotropic constructs not only induced stem cell alignment along the macroscale-organized scaffolds, but also produced 3D cell alignment along the microscale hierarchical MNP chains. Specifically, Traldi et al. developed an anisotropic hybrid construct by incorporating melt electrowritten PCL scaffolds with cell-laden GelMA hydrogels, but the anisotropic cellular network only exists on the surface of the axial fibers of the PCL scaffolds, not among the GelMA hydrogel [87]. Besides, Galindo et al. compared the melt electrowritten PCL scaffolds with/without RGD modified hyaluronic acid (HA) hydrogels, and found more cells attached and spread along the anisotropic HA-RGD-PCL scaffolds than HA-PCL scaffolds and PCL scaffolds alone [88]. Interestingly, although the HA hydrogels were coated on the whole scaffolds, there were no cells spread between two filaments of the scaffold, indicating the limited cell-matrix interactions in the HA hydrogels. Notably, in our PEA-COPMA-MNP hybrid constructs, we observed good cell attachment and spreading both on the scaffolds and in the hydrogels, without requiring any bioactive peptide sequence conjugation (e.g., RGD), likely due to the good bioactivity of COPMA hydrogels. Due to achieving alignment in both macroscale and microscale, hMSCs were aligned throughout the nanocomposite hybrid constructs.

In our study, the alignment and aggregation of MNPs are highly under control during the hydrogel crosslinking process. To improve MNP dispersion, we coated MNP with carboxylic acid, leading to a narrow polydispersity index. These MNPs were dispersed well in the hydrogel precursor and then assembled into head-to-tail MNP chains parallel to the magnetic field direction because of magnetic dipole–dipole attraction [89]. In the meanwhile, hydrogels were crosslinked under UV light, where MNP chains can be easily captured in the hydrogel network due to the non-covalent interactions between hydrogels and MNPs, further inhibiting particle aggregation. To better control MNP aggregation and alignment, future studies could focus on optimizing MNP concentration, MNP aspect ratio, magnetic field strength, and hydrogel curing time [90]. Long-term accumulation of MNPs in tissues and organs remains an important consideration for regenerative medicine applications. Chansoria et al. highlighted that magnetic biomaterials should consider nanoparticle size, biodegradability, biodistribution, and clearance behavior to reduce long-term bioaccumulation risks [91]. Thus, to increase clinic translation of aligned constructs, future studies may focus on optimizing MNP size, concentration and surface coating modification.

## 5 Conclusion

This study provided a hybrid strategy to fabricate mechanically reinforced 3D cellular networks with either macroscale anisotropy or dual macro-micro-scale anisotropy by incorporating collagen-derived hydrogels with uniaxial 3D printed amino acid-based PEA scaffolds. The PEA-COPMA hybrid scaffold showed macroscale anisotropy, flexibility and rapid UV-curing properties. The PEA-COPMA-MNP hybrid scaffold showed dual macro-micro-scale anisotropy and rapid dual light-magnetic responsiveness. These hybrid constructs maintained good integrity and biocompatibility over 28 days. Compared to the macroscale anisotropic constructs (PEA-COPMA), the PEA-COPMA-MNP hybrid constructs with dual macro-micro-scale alignment promoted continuous cellular alignment across the scaffold and hydrogel, rather than restricting alignment on the scaffold surface alone. In absence of exogenous biochemical cues supplementation (e.g., bioactive growth factors), collagen-derived constructs facilitated hMSCs differentiation in macroscale anisotropic constructs thanks to their intrinsic biochemical cues, with higher GAG, COL-I and SCX expressions on day 28 than on day 2. Furthermore, cell alignment and differentiation were further enhanced in the dual macro-micro-scale anisotropic nanocomposite constructs, due to the complementary effect of biophysical anisotropic cues and intrinsic biochemical cues. This hybrid biofabrication strategy offers a versatile route to fabricate mechanically robust and aligned constructs for engineering 3D *in vitro* tissue models with tunable multi-scale anisotropy, such as ligaments, tendons and muscles.

## CRediT authorship contribution statement

**Hongjuan Weng:** Conceptualization, Formal analysis, Funding acquisition, Investigation, Methodology, Writing – original draft, Writing – review & editing. **Lei He:** Formal analysis, Investigation, Methodology, Writing – review & editing. **Wen Chen:** Formal analysis, Investigation, Methodology, Writing – review & editing. **Vahid Ansari:** Formal analysis, Methodology, Writing – review & editing. **Sabine van Rijt:** Funding acquisition, Resources, Supervision, Writing – review & editing. **Monize C. Decarli:** Conceptualization, Methodology, Supervision, Writing – review & editing. **Katrien V. Bernaerts:** Conceptualization, Funding acquisition, Project administration, Resources, Supervision, Writing – review & editing. **Lorenzo Moroni:** Conceptualization, Formal analysis, Funding acquisition, Project administration, Resources, Supervision, Writing – review & editing.

## Declaration of competing interest

The authors declare that they have no known competing financial interests or personal relationships that could have appeared to influence the work reported in this paper.

## Data Availability

Data will be made available on request.
