# Supplementary material for: Elastic scaffolds reinforced stem cell-laden collagen-derived hybrid hydrogels to engineer 3D anisotropic cellular microenvironment
Source: Mater Today Bio. 2026 Jun 22;39:103385. doi: 10.1016/j.mtbio.2026.103385 (PMC13330686; doi:10.1016/j.mtbio.2026.103385)
Supplement: Multimedia component 1 [file mmc1.pdf]

Supporting information

Elastic scaffold reinforced stem cell-laden collagen-derived hybrid hydrogels to engineer 3D anisotropic cellular microenvironment

Hongjuan Weng[1,2], Lei He[3], Wen Chen[1], Vahid Ansari[1,2], Sabine van Rijt[3], Monize C. Decarli[1,4], Katrien V. Bernaerts[2*], Lorenzo Moroni[1*]

1 Complex Tissue Regeneration Department, MERLN Institute for Technology-Inspired Regenerative Medicine, Maastricht University, The Netherlands

2 Sustainable Polymer Synthesis Group, Aachen-Maastricht Institute for Biobased Materials, Maastricht University, The Netherlands

3 Instructive Biomaterials Engineering Department, MERLN Institute for Technology-Inspired Regenerative Medicine, Maastricht University, The Netherlands

4 Department of Biomaterials and Biomedical Technology, University Medical Center Groningen, University of Groningen, The Netherlands

Synthesis of monomer A

Monomer A (di-p-toluenesulfonic acid salt of bis(glycine)hexane 1,6-diester; Gly-E) was synthesized following the protocol reported by Ansari et al [1]. In brief, glycine (186.2 mmmol), 1,6-hexanediol (84.6 mmol), *p*-toluenesulfonic acid monohydrate (PTSA, 186.2 mmol), and 430 mL of dry toluene were combined in a 1 L three-necked flask. This setup was equipped with a nitrogen inlet, mechanical stirrer, thermometer, Dean-Stark apparatus, and reflux condenser fitted with a calcium chloride drying tube on the top. The reaction mixture was refluxed for 8 h until around 6.4 mL of water was collected in the Dean-Stark. Heating and stirring were then stopped, and the reaction mixture was cooled down gradually. The viscous, light yellowish product that settled at the flask bottom was decanted and washed twice with tert-butyl methyl ether under stirring. The resulting whitish solid was dried overnight in a vacuum oven at 40 °C to remove residual solvents. The monomer was dissolved in methanol/water (70/30, v/v) with heating and stirring until a clear solution was obtained. After stopping heating and stirring, the solution was recrystallized successively from methanol/water (70/30, v/v) until no impurity peak was observed in the ^{1}H NMR spectrum. The purified white crystalline product was vacuum dried sequentially at 40 °C for 24 h, 60 °C for 24 h, and 80 °C for 48 h. Yield: 78%.

Synthesis of monomer B

Monomer B (di-p-nitrophenyl adipate; NA) was synthesized following the protocol reported Ansari et al [1]. In brief, a solution of p-nitrophenol (329.5 mmol) and dry triethylamine (329.5

mmol) was mixed in 580 mL of dry acetone and cooled in an ice bath in a 1 L three-necked flask equipped with a calcium chloride drying tube. Separately, adipoyl chloride (163.9 mmol) was mixed with 58 mL of cool dry acetone and added dropwise via a dropping funnel to the stirring mixture. After stirring at around 0 °C for 2 h, the reaction mixture was stirring at room temperature overnight. The resulting mixture was precipitated into cold distilled water, and the solid product was collected by vacuum filtration using a Buchner funnel. The crude product was washed with distilled water and vacuum dried at 50 °C for 24 h. The product was further recrystallized seven times from acetonitrile, yielding off-white crystals after gradual drying under vacuum at 60–100 °C for 48 hours. Yield: 87%.

Characterizations of PEA

The synthesis of PEA was verified by ^{1}H NMR spectroscopy (Bruker Avance III-HD, 300 MHz, Germany) with DMSO-d_6 as solvent. Molecular weights and dispersity of the PEA were determined by gel permeation chromatography (GPC) using a PSS system (Agilent Technologies 1260 Infinity, USA) equipped with refractive index (RI) detector and two PFG combination medium columns plus a PFG precolumn (particle size: 5 µm, Agilent). Analyses were performed using 1,1,1,3,3,3-hexafluoroisopropanol (99.9% purity, EVOCHEM Advanced Materials) containing 0.019% sodium trifluoroacetate (98% purity, Sigma-Aldrich) as the eluent at a flow rate of 0.33 mL/min, with poly(methyl methacrylate)s (PMMA) standards for calibration. The thermogravimetric analysis (TGA) was conducted on a TA Instruments Q500 thermogravimetric analyzer (Waters, USA) under nitrogen, heating from 25–700 °C at 10 °C/min. Differential scanning calorimetry (DSC) analyses were performed using a Netzsch DSC214 Polyma (Germany) under nitrogen flow (20 mL/min) with two heating/cooling cycles between -40 and 200 °C at 10 °C/min. The glass transition temperature was determined from the midpoint of the transition. The melting point of the PEA was obtained from the peak of second heating cycle.

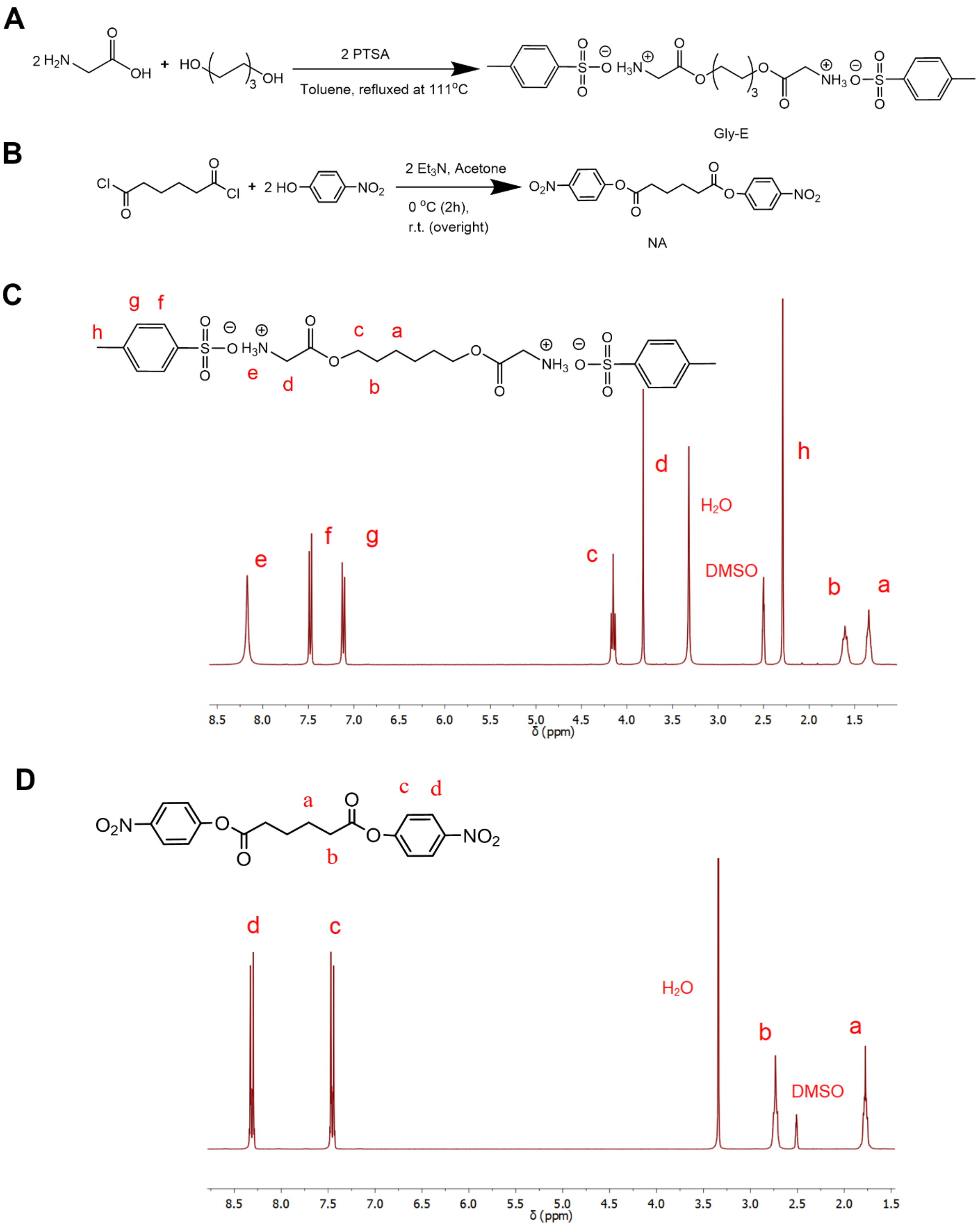

Fig. S1. Synthesis of monomers. A-B) Reaction scheme of Gly-E and NA. C) ^{1}H NMR spectrum of Gly-E. The peaks labeled *a*–*h* were assigned to the corresponding hydrogen atoms of Gly-E: *a* at 1.34 ppm (-$\underline{CH_2}$-CH_2-CH_2-OCO-), *b* at 1.61 ppm (-CH_2-$\underline{CH_2}$-CH_2-OCO-), *c* at 4.15 ppm (-CH_2-

CH_2-$\underline{CH_2}$-OCO-), *d* at 3.82 ppm (-OCO-$\underline{CH_2}$-NH_3^+-), *e* at 8.18 ppm (-$\underline{NH_3^+}$), *f* at 7.46 ppm (-$\underline{Ph}$-CH_3), *g* at 7.10 ppm (-SO_3^--$\underline{Ph}$-), and *h* at 2.29 (-Ph-$\underline{CH_3}$). D) ^{1}H NMR spectrum of NA. The peaks labeled *a–d* were assigned to the corresponding hydrogen atoms of NA: *a* at 1.77 ppm (-OCO-CH_2-$\underline{CH_2}$-), *b* at 2.73 ppm (-OCO-$\underline{CH_2}$-CH_2-), *c* at 7.44 ppm (-O-$\underline{Ph}$-), and *d* at 8.3 ppm (-$\underline{Ph}$-NO_2).

A

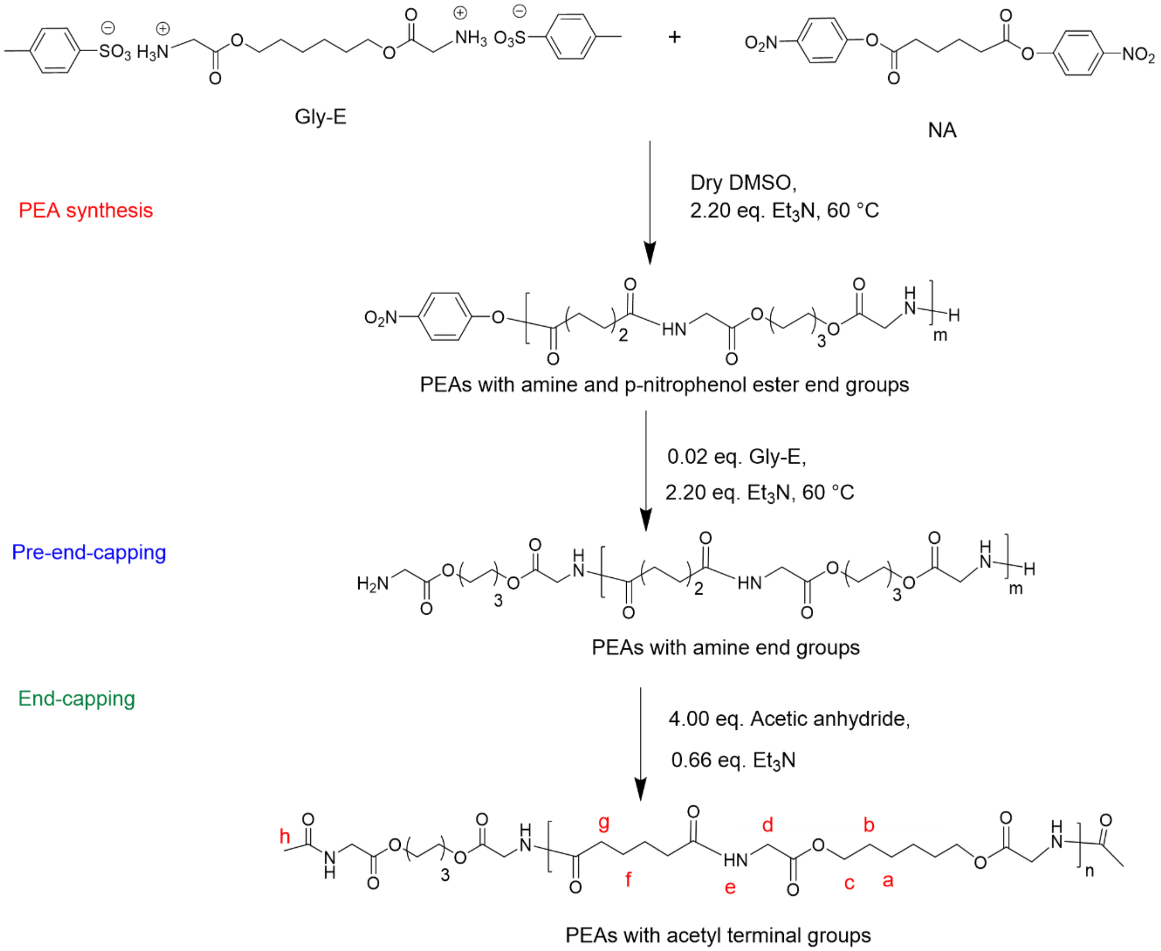

Gly-E
NA
PEA synthesis
Dry DMSO,
2.20 eq. Et3N, 60 °C
PEAs with amine and p-nitrophenol ester end groups
0.02 eq. Gly-E,
2.20 eq. Et3N, 60 °C
Pre-end-capping
PEAs with amine end groups
End-capping
4.00 eq. Acetic anhydride,
0.66 eq. Et3N
PEAs with acetyl terminal groups

B

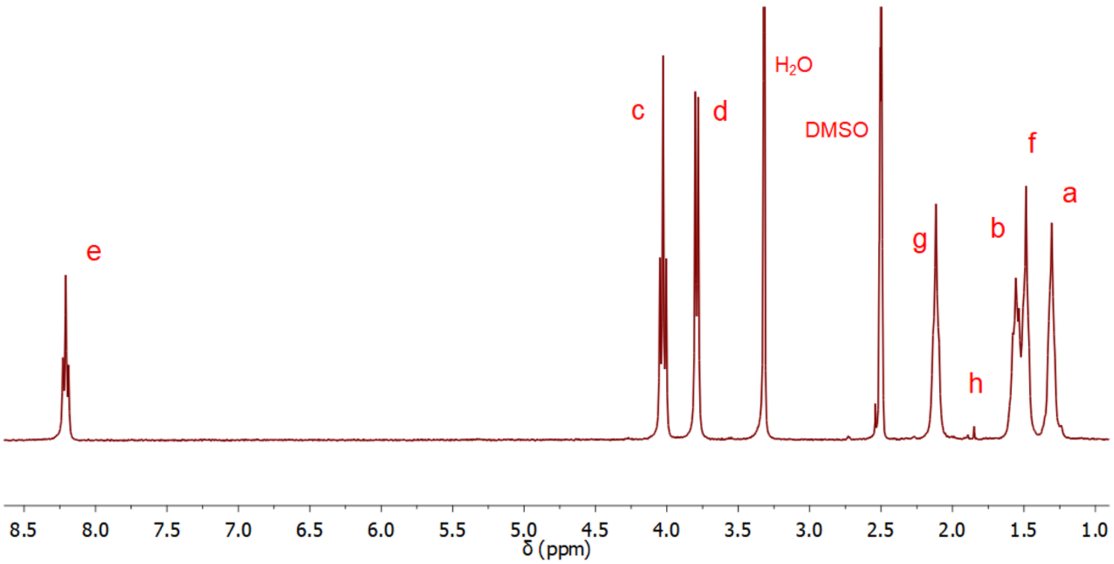

e
c
d
H2O
DMSO
g
h
b
f
a
8.5 8.0 7.5 7.0 6.5 6.0 5.5 5.0 4.5 4.0 3.5 3.0 2.5 2.0 1.5 1.0
δ (ppm)

Fig. S2. Synthesis of PEA. A) Reaction scheme. B) ^{1}H NMR spectrum of PEA. The peaks labeled *a–h* were assigned to the corresponding hydrogen atoms of PEA: *a* at 1.33 ppm (-COOCH_2C$H_2\underline{CH_2}$-), *b* at 1.56 ppm (-COOC$\mathit{H}_2\underline{CH_2}CH_2$-), *c* at 4.01 ppm (-COO$\underline{C\mathit{H}_2}C\mathit{H}_2C\mathit{H}_2$-), and *d* at 3.80 ppm (-NH$\underline{CH_2}$COOCH_2-), *e* at 8.23 ppm (-NH-), *f* at 1.49 ppm (-NHCOC$\mathit{H}_2\underline{C\mathit{H}_2}$-), *g* at 2.12 ppm (-NHCO$\underline{C\mathit{H}_2}C\mathit{H}_2$-), and *h* at 1.85 ppm (-CH_3).

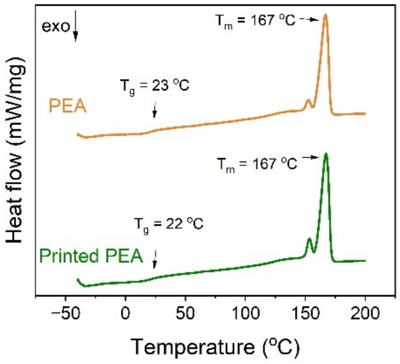

Fig. S3. DSC curve of PEA and printed PEA. The T_g and T_m values of printed PEA remained nearly unchanged after FDM printing, indicating that the printing process did not significantly affect the molecular mobility or crystal structure of the polymer. This suggested that PEA could be reprinted into scaffolds using the same printing temperature (185 °C) as that used for the initial PEA scaffold fabrication.

Table S1. Molecular weight and thermal properties of PEA.

	M_n (kg/mol)	Đ	$T_{5\%}$ (°C)	T_g (°C)	T_m (°C)	T_c (°C)
PEA	44.6	2.3	342	23	167	127
Printed PEA	36.4	2.8	346	22	167	129

Table S2. Optimized printing parameters of PEA scaffolds based on printhead sepeed and screw speed.

Sample	Printhead speed (mm/min)	Screw speed (R/min)
PEA scaffold-1	400	100
PEA scaffold-2	300	100
PEA scaffold-3	300	2000
PEA scaffold-4	300	3000
PEA scaffold-5	200	2000

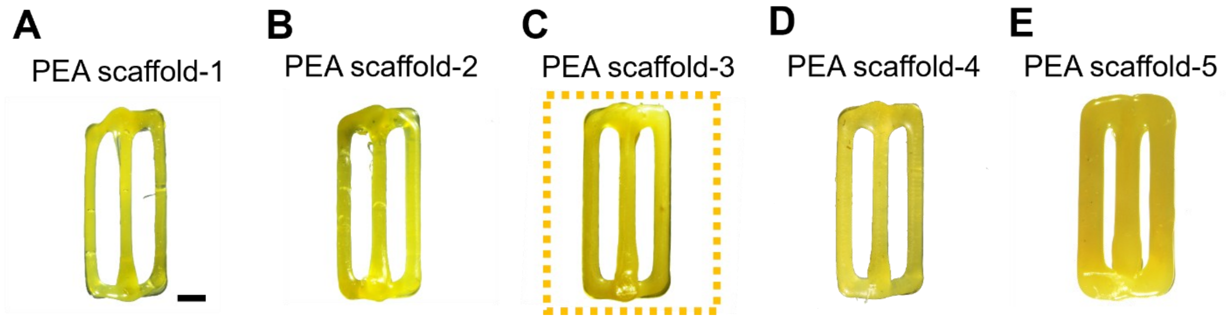

Fig. S4. Optical image of PEA scaffolds with different 3D printing parameters, scale bar: 1000 μm. The orange frames indicate the optimized PEA scaffolds printed by optimized parameters.

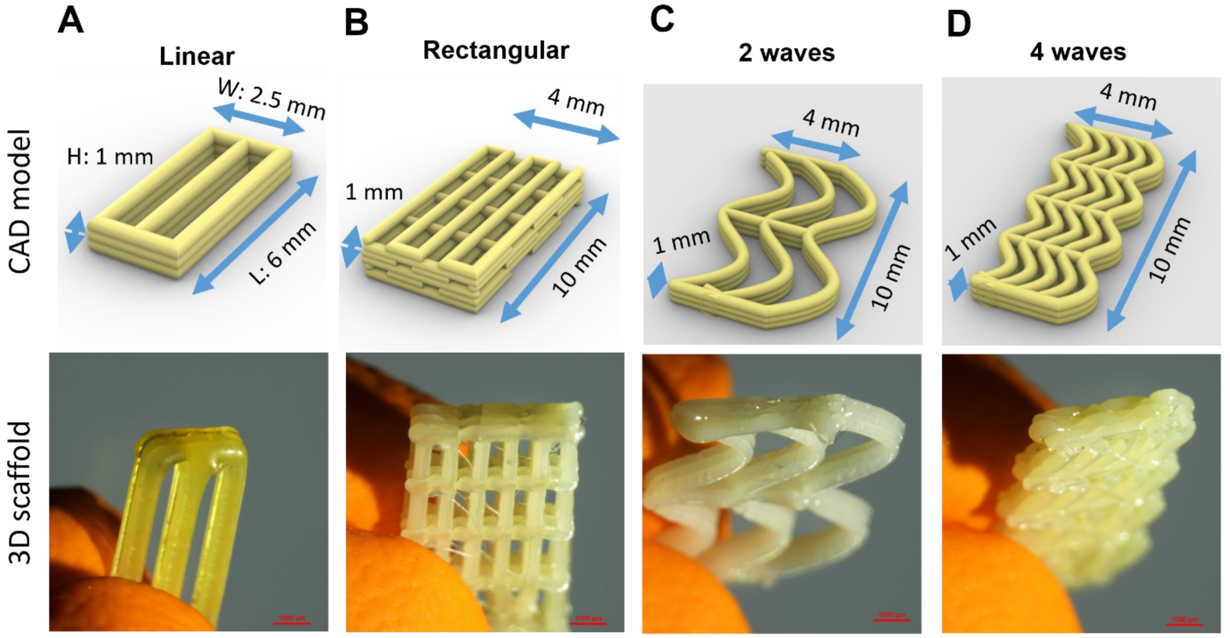

	Theoretical parameters (mm)			Practical parameters (mm)		
	Length	Width	Hight	Length	Width	Hight
Linear	6	2.5	1	6.59±0.15	3.00±0.05	1.30±0.08
Rectangular	10	4	1	10.64±0.06	4.48±0.08	1.09±0.06
2 waves	10	4	1	10.47±1.00	4.79±1.00	0.93±0.06
4 waves	10	4	1	10.63±0.04	4.18±0.04	1.06±0.03

Fig. S5. CAD models and the represent pictures of 3D printed scaffolds in different geometries. A) Linear scaffold. B) Rectangular scaffold. C-D) Wavy scaffolds. Scale bar: 1 mm. The 3D printed scaffolds showed high similarity to the CAD models in terms of morphology, length, width and height.

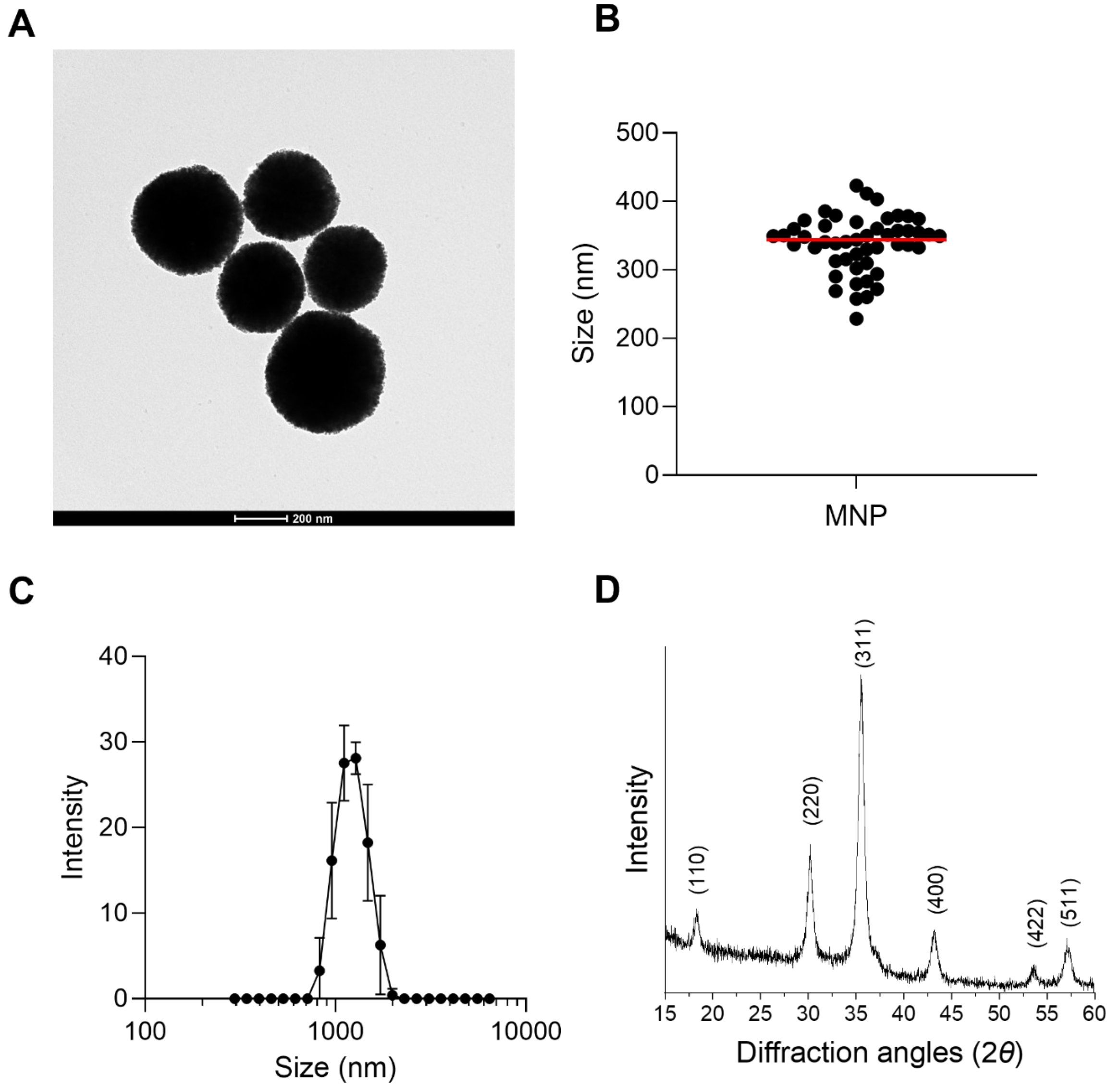

Fig. S6. Characterization of MNPs. A) TEM image of MNPs, scale bar: 200 nm. B) Size distribution of MNPs based on TEM images. C) Hydrodynamic size of MNPs analyzed by DLS. D) XRD spectrum of MNPs.

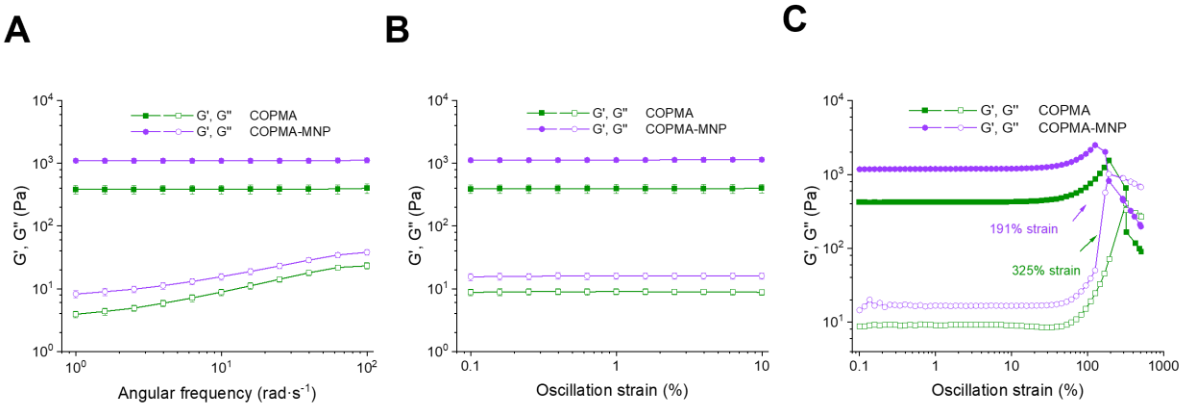

Fig. S7. A) Rheological measurements of COPMA and COPMA-MNP hydrogels in frequency sweeps from 1 to 100 rad·s^{-1}, with a constant strain of 2%. B) Strain sweep mode from 1 to 10% with a constant frequency of 10 rad·s^{-1}. C) Strain sweeps of COPMA and COPMA-MNP hydrogels from 0.1% to 500% at an angular frequency of 10 rad·s^{-1}.

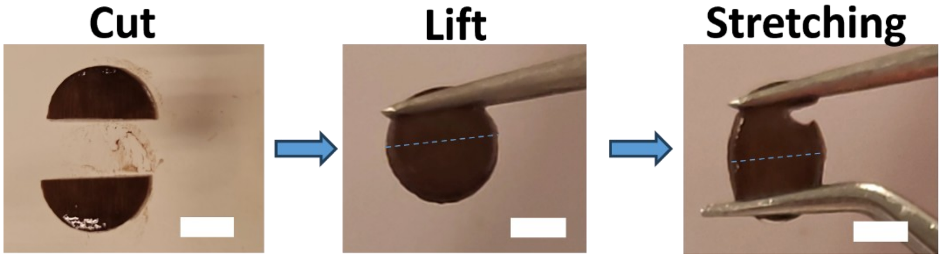

Fig. S8. Self-healing process of COPMA-MNP hydrogel, scale bar: 4 mm. Blue lines indicate the rupture position before self-healing. Two cut COPMA-MNP hydrogels could be rejoined at room temperature for 3 hours. The self-healed hydrogel could sustain gravity and a certain strength without interfacial separation.

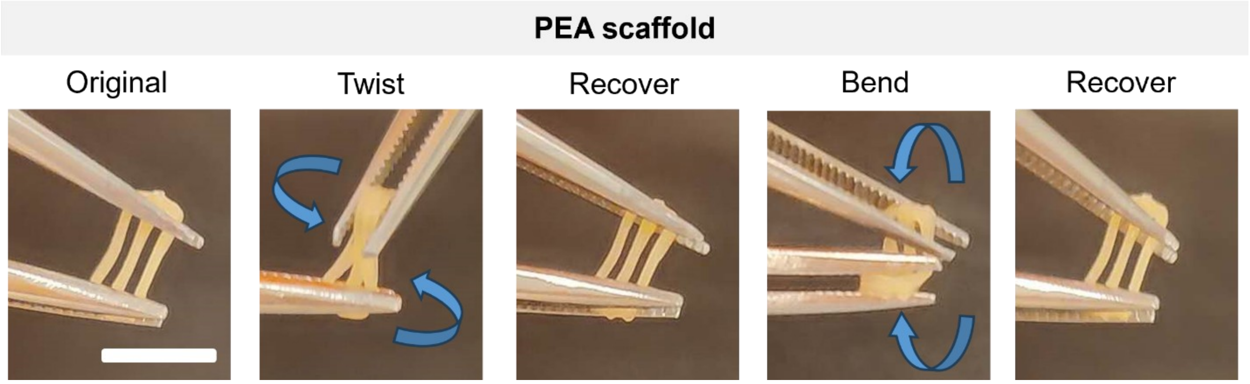

Fig. S9. Elasticity of PEA scaffolds, scale bar: 6 mm.

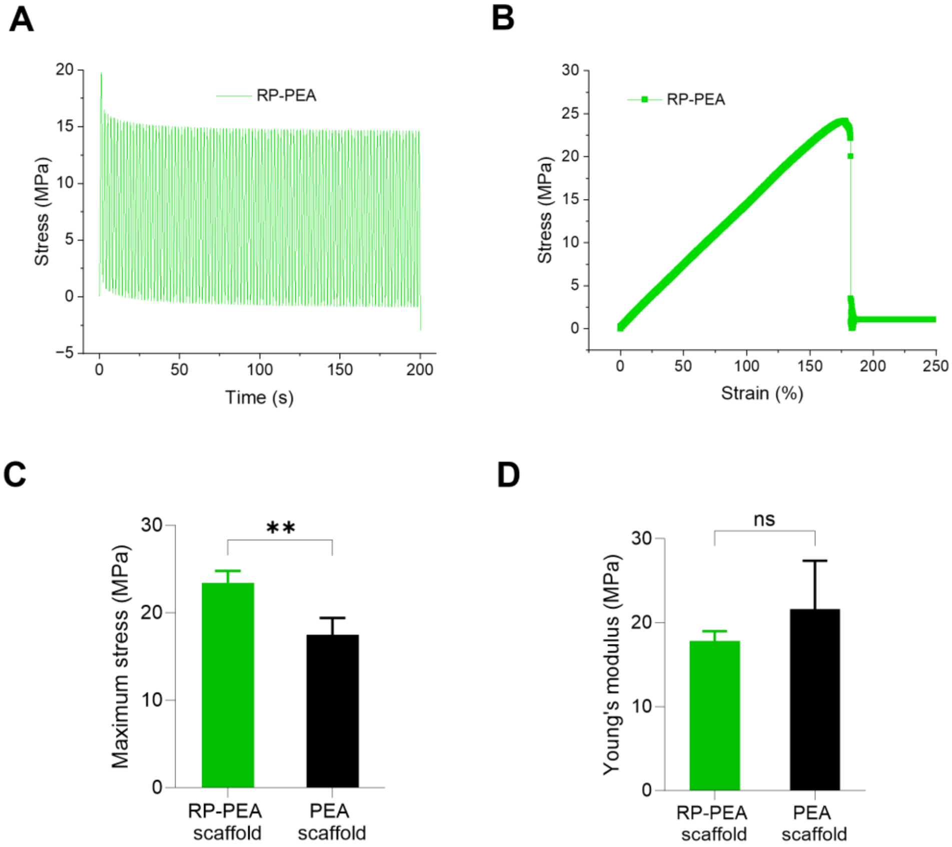

Fig. S10. Tensile test of RP-PEA scaffolds. A) Cyclic tensile tests of RP-PEA scaffolds at 10% strains for 100 cycles. B) Tensile stress-strain curves of RP-PEA scaffolds. C) Comparison of maximum tensile stress of RP-PEA scaffolds and PEA scaffolds. D) Comparison of young's modulus of RP-PEA scaffolds and PEA scaffolds ($n \geq 3$, ns indicates no significant difference, $**p < 0.01$).

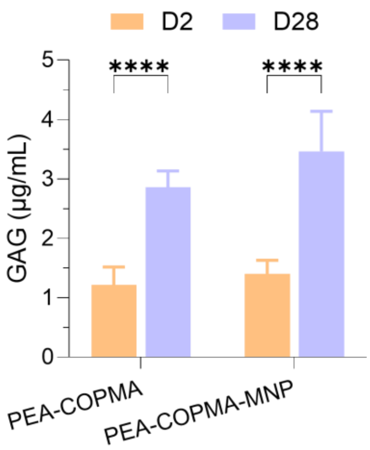

Fig. S11. GAG production in PEA-COPMA and PEA-COPMA-MNP hybrid constructs on day 2 and day 28 (n ≥ 3, ****$p < 0.0001$).

Reference:

[1] V. Ansari, A. Calore, J. Zonderland, J. A. W. Harings, L. Moroni, K. V. Bernaerts, *Biomacromolecules* **2022**, 23, 1083.
